# A Liftless Intervention to Prevent Preterm Birth and Low Birthweight Among Pregnant Ghanaian Women: Protocol of a Stepped-Wedge Cluster Randomized Controlled Trial

**DOI:** 10.2196/10095

**Published:** 2018-08-23

**Authors:** Emma Kwegyir-Afful, Jos Verbeek, Lydia Aziato, Joseph D Seffah, Kimmo Räsänen

**Affiliations:** ^1^ Institute of Public Health and Clinical Nutrition School of Medicine University of Eastern Finland Kuopio Finland; ^2^ Finnish Institute of Occupational Health Kuopio Finland; ^3^ School of Nursing and Midwifery University of Ghana Accra Ghana; ^4^ Department of Obstetrics & Gynaecology University of Ghana Medical School Accra Ghana

**Keywords:** heavy lifting, Ghana, low birthweight, liftless intervention, low-and middle-income countries, physical activity, preterm birth, randomized controlled trial, stepped-wedge

## Abstract

**Background:**

Preterm birth (PTB) is a leading cause of infant morbidity and mortality worldwide. Every year, 20 million babies are born with low birthweight (LBW), about 96% of which occur in low-income countries. Despite the associated dangers, in about 40%-50% of PTB and LBW cases, the causes remain unexplained. Existing evidence is inconclusive as to whether occupational physical activities such as heavy lifting are implicated. African women bear the transport burden of accessing basic needs for their families. Ghana’s PTB rate is 14.5%, whereas the global average is 9.6%. The proposed liftless intervention aims to decrease lifting exposure during pregnancy among Ghanaian women. We hypothesize that a reduction in heavy lifting among pregnant women in Ghana will increase gestational age and birthweight.

**Objective:**

To investigate the effects of the liftless intervention on the incidence of PTB and LBW among pregnant Ghanaian women.

**Methods:**

A cohort stepped-wedge cluster randomized controlled trial in 10 antenatal clinics will be carried out in Ghana. A total of 1000 pregnant participants will be recruited for a 60-week period. To be eligible, the participant should have a singleton pregnancy between 12 and 16 weeks gestation, be attending any of the 10 antenatal clinics, and be exposed to heavy lifting. All participants will receive standard antenatal care within the control phase; by random allocation, two clusters will transit into the intervention phase. The midwife-led 3-component liftless intervention consists of health education, a take-home reminder card mimicking the colors of a traffic light, and a shopping voucher. The primary outcome are gestational ages of <28, 28-32, and 33-37 weeks. The secondary outcomes are LBW (preterm LBW, term but LBW, and postterm), compliance, prevalence of low back and pelvic pain, and premature uterine contractions. Study midwives and participants will not be blinded to the treatment allocation.

**Results:**

Permission to conduct the study at all 10 antenatal clinics has been granted by the Ghana Health Service. Application for funding to begin the trial is ongoing. Findings from the main trial are expected to be published by the end of 2019.

**Conclusions:**

To the best of our knowledge, there has been no randomized trial of this nature in Ghana. Minimizing heavy lifting among pregnant African women can reduce the soaring rates of PTB and LBW. The findings will increase the knowledge of the prevention of PTB and LBW worldwide.

**Trial Registration:**

Pan African Clinical Trial Register (PACTR201602001301205); http://apps.who.int/trialsearch/ Trial2.aspx?TrialID=PACTR201602001301205 (Archived by WebCite at http://www.webcitation.org/71TCYkHzu)

**Registered Report Identifier:**

RR1-10.2196/10095

## Introduction

Preterm birth (PTB) and low birthweight (LBW) are increasing worldwide, especially within low-and middle-income countries (LMIC). Every year, 15 million babies are born before 37 completed weeks [1,2] and 20 million (15.5%) babies are born with LBW (˂2500 g) worldwide. Approximately 96% of these births occur in LMIC [1]. It is estimated that LBW infants are at a 20-fold greater risk of neonatal mortality than babies with a birthweight of 3.5-4.0 kg. Approximately 28% of neonatal deaths (first 7 days of life) are a result of PTB [3]. PTB is a major contributing factor to LBW [1]. Millions of preterm and LBW babies die as a result of preventable complications, and some of those who survive will live with lifelong debilitating health conditions [1,4,5]. PTB is a problem even in high-income countries (HIC). The rate of PTB in the United States increased from 9.5% in 1981 to 12.7% in 2005 [6]. The rate of PTB averages 9.6% worldwide and varies between Europe (6.2%) and North America (10.6%) [6]. In LMIC, the incidence of LBW is around 19%, compared with 5%-7% in HIC [7]. Southern Asia and sub-Saharan Africa have recorded 60% of global PTBs in 2010; Ghana was ranked 14th of 184 countries, with a PTB rate of 14.5% in 2010 [2].

There is widespread agreement on the need for immediate action to prevent PTB and LBW. However, this cannot be completed without a critical look at the occupational environment of the ever-increasing global female workforce [8]. A greater number of women in LMIC work in the informal sector [9], and their work entails repetitive lifting and carrying [10] with virtually no ergonomic guidelines to safeguard their welfare. Evidence suggests that most African women perform an average of 328 trunk flexions at angles exceeding 60º in an 8-hour period each day. Of these trunk flexions, 66 are sustained for >4 seconds [11]. In household settings, African women bear most of the transport burden of accessing basic needs such as water, farm produce, and firewood. The average African woman carries a load of about 20 kg over a distance between 2.5 and 6.8 km on a daily basis [11]. The physical stress encountered by these women can result in musculoskeletal disorders and negative reproductive consequences [12-14]. The activities of ordinary Ghanaian women in rural and periurban areas include farming, carrying water and farm produce on their heads, carrying market wares during street hawking, and carrying their younger children on their backs (at home, to the market, and to the farm).

Some observational studies have implicated occupational lifting or heavy physical workload during pregnancy in the causal pathways of PTB and LBW [1,12,15-17], although others did not reach clear conclusions [18]. However, exposure may have been poorly measured, thereby distorting the outcome of such studies. The Occupational Health Clinic for Ontario Workers reported that a 4.5 kg weight carried further away from the back during pregnancy exerts 68 kg of stress on the lower back, compared with 29.5 kg of stress in a nonpregnant woman [19]. A rise in intra-abdominal pressure resulting from lifting can initiate premature uterine contractions [10], and lifting increases the risk of pelvic pain during gestation [20].

There have been many successful health education and advice-oriented interventions that have improved patient outcomes [21]. In their quest to lengthen gestational age, midwives in LMIC such as Ghana advise pregnant women against strenuous physical activity. In certain conditions, the pregnant woman is admitted to a hospital throughout the gestational period to ensure complete bed rest to avert possible PTB [22]. Several occupational guidelines have been formulated by various institutions [19,23] aimed at protecting pregnant employees against physical stress in their respective countries. More recent are the provisional clinical guidelines for occupational lifting in pregnancy by MacDonald et al [24]. However, no such guidelines exist in Ghana and other LMIC. A recent systematic review revealed that no trial has investigated the usefulness of such guidelines aimed at reducing heavy lifting among pregnant women to increase gestational age [22]. The 3-component liftless intervention implemented in this trial is based on the clinical guidelines of occupational lifting in pregnancy by MacDonald et al [24] coupled with a shopping voucher. The rationale for the shopping voucher is to augment the uptake of the intervention components [25,26]. The shopping voucher will be administered during the third trimester with the intention of reducing moderate PTB, which is common in sub-Saharan Africa [2]. As no randomized controlled trial (RCT) has been conducted to ascertain the effectiveness of the proposed liftless intervention, the outcomes will be of public health importance for elucidating the effects of heavy lifting on birth outcomes (PTB and LBW). Through this study, a model for intervention to reduce physical exertion during pregnancy will be developed and its impact on reducing PTB and LBW will be ascertained. The success of the proposed stepped-wedge trial will justify the need for clinical guidelines to modify the occupational and family environment of pregnant women in LMIC. We hypothesize that a reduction in heavy lifting among pregnant women in Ghana will increase gestational age and birthweight.

The objective of this study is to investigate the effects of the liftless intervention on the incidence of PTB and LBW among pregnant Ghanaian women. This study aims (1) to provide guideline-oriented and social support (liftless intervention) to Ghanaian women to reduce their lifting behavior during pregnancy; (2) to evaluate the effect of the liftless intervention on rates of PTB, LBW, mode of delivery, lower back or pelvic pain, and premature uterine contractions compared to no such intervention; and (3) to examine compliance with the intervention and ascertain factors that influence it.

## Methods

### Study Design

**Figure 1:**
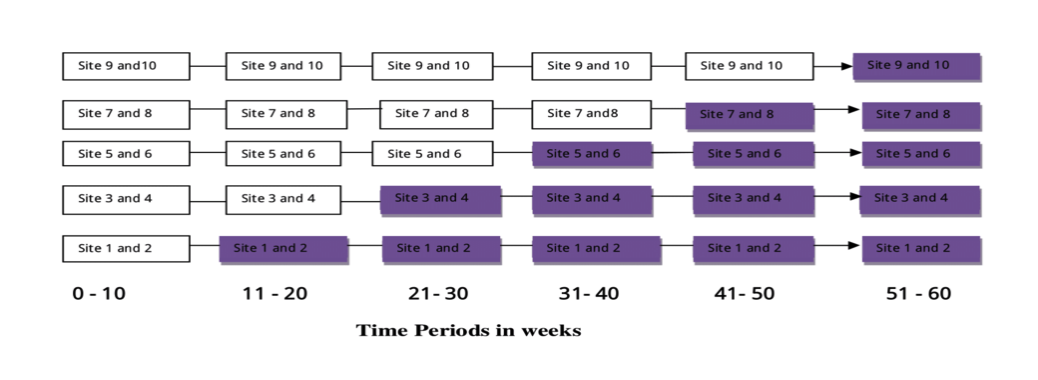
Stepped-wedge design. Shaded boxes: intervention phases; white boxes: control phases. (Adopted from a trial protocol by Hill AM, Waldron N, Etherton-Beer C, et al 2014).

**Figure 2:**
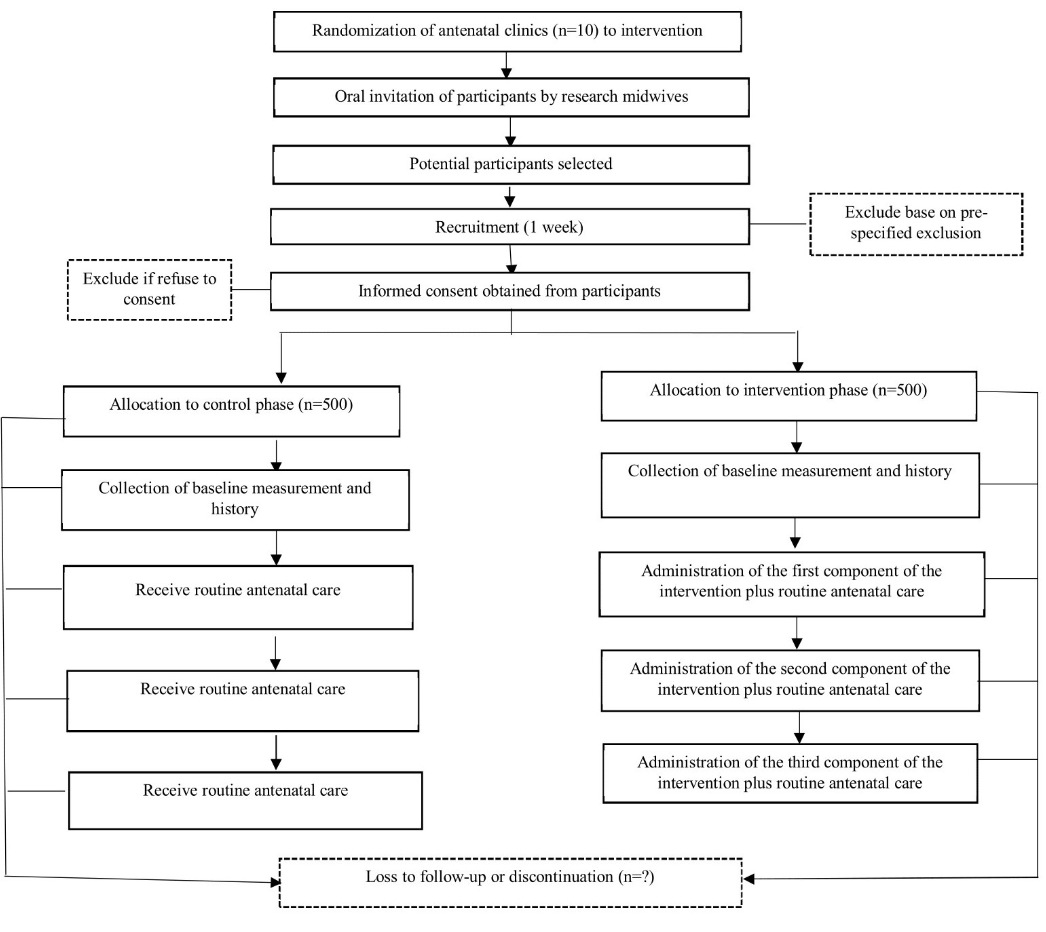
Flow of the trial (adopted from Standard Protocol Item: Recommendations for International Trials 2013).

We propose an open cohort stepped-wedge cluster RCT (PACTR201602001301205). In this design, eligible participants will be recruited until the expected numbers have been achieved. The design is a unidirectional cross-over study wherein all participating antenatal clinics will start with a control phase and eventually end up providing the intervention. The intervention will not be withdrawn once implementation has begun (at least not until the end of the trial). Participants will leave the trial when they deliver. Baseline measurements and information will be collected in all 10 clusters at the beginning of the trial using a 55-item data collection tool. Using random allocation, two clusters will transition into the intervention with each phase lasting 10 weeks. With the exception of clusters 1 and 2 (Figure 1) [27], the rest of the clusters will contribute data to the control more than once. Clusters 9 and 10 will contribute control data at 5 time points before the intervention is implemented. Thus, unlike a traditional cluster RCT where the intervention is administered only to those in the treatment group, every cluster in the stepped-wedge RCT will receive the intervention. The flow of the trial is shown in Figure 2 [28].

During the control phases, participants will receive routine antenatal care including physical and abdominal examination, blood pressure and weight measurement, urine testing, prescription of iron and folic acid supplementation, and tetanus injection. When a clinic enters the intervention phase, in addition to receiving routine antenatal care, participants will attend 5 extra intervention sessions at weekly intervals. During the sessions, study midwives will deliver the 3-component liftless intervention explaining and demonstrating all of the potential harmful task conditions, providing a take-home reminder card, and soliciting for a partner’s support. In addition, midwives will make phone calls, do home visits, and provide the shopping vouchers.

The choice of the stepped-wedge design for this trial is based on the anticipation that the intervention will be useful. This design allows the intervention to be offered to all clusters, reduces potential contamination of the control group [29], and provides an opportunity to gradually roll out the intervention to resolve any financial or logistical constraints that may arise at the clinics concurrently. To ensure completeness of the protocol, the Standard Protocol Item: Recommendations for International Trials 2013 recommendation was followed [28].

### Study Population and Recruitment

The trial will be conducted at 10 public antenatal clinics in Ghana (the lists of clinics are provided in Multimedia Appendix 1). Eligible clinics will be those that offer antenatal services. The selected clinics are located in 4 cities in Ghana. The clinics will recruit 1000 participants within a period of 60 weeks. Although the facilities vary in size, they are located in areas that have sufficient numbers of pregnant women to make the trial feasible. The socially ascribed activities of most women within the catchment areas of the proposed antenatal clinics include carrying water and farm produce on their heads as well as carrying market wares as street vendors. Most women also carry their younger children on their backs at home, to the market, and to the farm. Additional antenatal clinics will be added to meet the planned target if necessary. The study midwives will recruit participants based on the prespecified participant-level criteria (Textboxes 1 and 2). The nature of the trial and the required commitment on the part of participants will be explained to the participants by the research midwives in a language that they understand. Participation is voluntary and decisions will not affect the routine antenatal care they will receive. Those who agree to participate and are eligible will be provided with a consent form to sign or thumb print (the copy of consent form is available on request from the authors).

### Outcomes

#### Primary outcome

The primary outcome is gestational age based on ultrasound examination in the first trimester (before 16 weeks), or when not available, fundal height and last menstrual period. We expect that only about 10% of the pregnancies will be without ultrasound diagnosis. We will still include these to prevent increasing the workload of the recruiting midwives. We will conduct a sensitivity analysis to see if there is an effect of the ultrasound versus last menstrual period and fundal height diagnosis. We will categorize PTB as extremely preterm (<28 weeks), very preterm (28-32 weeks), or moderate to late preterm (33-37 weeks) based on the World Health Organization classification [30].

#### Secondary outcomes

The secondary outcomes are as follows:

- Birthweight categorized as preterm LBW, full term but LBW, or postterm LBW babies [7].
- Compliance with the intervention (classified as compliant or noncompliant). We will classify a participant as compliant (1) if the weight and frequency of lifting have decreased to below the provisional guidelines and (2) if lifting below or above the knee or shoulders has decreased, based on the participant’s self-reported lifting and observations during home and workplace visits.
- Prevalence of lower back pain or pelvic pain: yes or no; frequency: sometimes or most of the time; stage of pregnancy: first or second or third trimester; severity: mild or moderate (interferes with daily function or severe and needs analgesics to subside).
- Premature uterine contractions: yes or no; frequency: sometimes or most of the time; stage of pregnancy: first or second or third trimester.
- Mode of delivery: normal or forceps or cesarean section, as recorded on birth and postnatal records.

**Inclusion criteria.**

Inclusion criteria

1. Pregnancy has been clinically confirmed.

2. Pregnancy is between 12 and 16 weeks.

3. Pregnancy is singleton.

4. Exposed to lifting of 10 kg at home or work.

5. Attending 1 of 10 antenatal clinics.

6. Participant has consented to participate in the trial.

**Exclusion criteria.**

Exclusion criteria

1. Pregnancy has not been confirmed.

2. Gestational age is above 16 weeks.

3. Multiple pregnancies.

4. Not exposed to lifting.

5. Not attending any of the selected clinics.

6. Refusal to participate.

### Randomization and Blinding

Using a computer-generated number sequence, the 10 antenatal clinics will be randomized as to when the intervention is implemented. To ensure internal validity of the study, the randomization will be carried out by a professional who is not involved in the delivery of the treatment or its assessment [31]. This will be done before the trial begins. However, due to the nature of the project, neither the research midwives nor participants can be blinded to the treatment allocation. However, a data analyst who will perform the final analysis will be blinded to the treatment allocation.

### Intervention

To encourage behavioral changes in heavy lifting tendencies among pregnant women, the study midwives will deliver the proposed 3-component liftless intervention with the assumption that increasing knowledge will promote behavioral changes even in the face of obstacles [32].

#### Component 1: Health Education

In this component, midwives will deliver health education based on the clinical guidelines for occupational lifting in pregnancy by MacDonald et al [24]. Topics will include possible implications of heavy lifting during pregnancy, avoiding lifting any object that weighs >10 kg, avoiding lifting items over shoulder height, dividing bigger objects into smaller portions before lifting, avoiding one-handed lifting, and avoiding lifting or lowering objects below the knee. Three boosters of this component will be given, and in addition, participants will be asked to invite their spouses or significant others to attend the next session. The aim is to solicit their support throughout gestation to boost compliance with the intervention. The duration for each session will be between 60 and 90 minutes.

At the end of the session, participants will be given a lifting or carrying diary to record their daily lifting exposure. Those who cannot read or write will be provided with an alternative means, such as making ticks on a paper or asking a relative to record the information, depending on the preferred option of the participant. The diary will be checked and the contents recorded at every session. The booklet will also contain the contact information of the midwives for participants to call whenever necessary.

#### Component 2: Take-home Reminder Card

This component consists of simplified explanations on a reminder card mimicking the colors of a traffic light, Red (SEEK), Yellow (STOP), Green (HOW), based on a similar trial carried out by Lumley and Donohue [33] in Melbourne. This component will be delivered at the second session and will last between 30 and 60 minutes.

##### SEEK (Red)

- A midwife’s assistance when you have questions or problems.
- A midwife’s assistance when you have severe lower abdominal pain, lower back pain, severe contractions, or vaginal bleeding or leaking of clear fluid (a more detailed explanation in a local language will be given).

##### STOP (Yellow)

- Lifting heavy loads that weigh >10 kg, either at home or at work.
- Lifting more than once per 5 minutes after pregnancy has been confirmed until term.
- Lifting or lowering objects below the knee.
- Lifting items over shoulder height.

##### HOW (Green)

- Divide objects into smaller portions before lifting.
- Attend the antenatal clinic and intervention sessions as scheduled.
- Seek help from close relatives when there is the need to lift an object weighing >10 kg.
- If possible, attend intervention sessions with a significant other.

#### Component 3: Shopping Voucher

The main focus of the third component will be the third trimester, as moderate preterm births (32 to <37 weeks) accounted for 84% of PTB in 2010, especially in sub-Saharan Africa [2]. This component is aimed to augment the uptake of the other components, as evidence has shown that voucher programs improve the efficiency and health of populations [25,26]. Participants will receive a shopping voucher equivalent to 40 GH₵ (about US $13 or 10€) at 6 different occasions beginning at the third session. The administration of the 6 shopping vouchers will span a period of 6 weeks from 32 to 37 weeks of gestation. The administration of the shopping voucher will begin with prearrangement of shops that sell items such as charcoal, water, and liquefied petroleum gas. Therefore, depending on the particular needs of each participant, they will receive a voucher to access the item from the preselected outlets, and the items will be delivered to participants at home.

Boosters of the first and second components will be delivered during the third and fourth sessions and will last between 30 and 60 minutes.

### Follow-up Telephone Call

As part of the intervention, research midwives will make telephone contact with participants after each intervention session to ascertain what measures they are taking to ensure compliance. Participants will be reminded to attend a session with their spouses or any significant family member days before a session.

### Home Visits

Research midwives will make 2 home visits (at the beginning and end of the study) with participants’ consent. The purpose is for midwives to objectively observe participants in their home environment to ascertain compliance and echo the content of the 3 components using a standardized observational checklist. The visits will also afford participants and their significant others an opportunity to seek clarification on any matters that they do not understand.

### Data Collection Tools and Data Management

First, a 55-item questionnaire will be used to collect sociodemographic data, baseline information on gestational age, family support, history of work activities, exposure to heavy lifting or carrying, lifting below the knee or above the shoulders, frequent bending, daily hours of standing, and repetitive movement. Second, the lifting and carrying diary will be used to assess participants’ self-reported daily frequency and weight of lifting and carrying and compliance. Third, participants’ antenatal and postnatal cards will be used to assess gestational age, birthweight, mode of delivery, and history of premature uterine contractions. The cards are usually filled out by midwives who provide antenatal care and deliver the child. Finally, the intervention session booklet will be used to record session attendance, participants’ complaints, and summary of education given. Any text data generated during home visits and telephone calls to or from the participants will also be recorded.

Data and materials obtained during the trial will be transferred and stored electronically, with a back-up on two memory sticks under the supervision of the primary researcher. The digital data will be disposed after dissemination and publication of the results.

### Statistical Analysis and Sample Size

We will compare the average proportion of all outcomes in the intervention group clusters to the control group clusters. We will test our hypothesis using a logistic regression model with a random effect for cluster and a fixed effect for each step of implementation of the intervention to adjust for possible effects of calendar time [34,35].

If there are major baseline differences between the intervention and control clusters for potential confounders, such as prolonged standing or squatting, parity, maternal age, infection, occupation, educational level, marital status, and nutritional status, we will include these as covariates in the model. Intention to treat will be the basis for the statistical analysis to ensure that all clusters (with participants) are randomized and included in the final analysis regardless of completion of the study or not [36].

For the sample size calculation, we set alpha at 0.1 and beta at 0.8. We assumed that the intervention will decrease PTB rate by 30% (ie, from 14.5% to 10.5%, with 14.5% being the current official PTB rate in Ghana). With 10 antenatal clinics, we estimated that we need 1000 participants to provide the power to detect a 30% difference in the rate of PTB between the control and intervention clusters. The individual performing the analysis will be blinded to the treatment allocation.

### Planning and Training

Prior to the start of the trial, the administrative heads of the selected antenatal clinics and midwives will be consulted. The rationale of the trial, contents of the intervention, periods of the trial, ability to perform the trial without disrupting the routine work of the participating midwives, and the recruitment process will be discussed. In consultation with the heads of the antenatal clinics, 30 research midwives will be selected. All participating midwives will receive trial-specific training using appropriate training methods by the first author. The training will be group-based to ensure consistency in the delivery of the intervention and will include how to recruit participants using the inclusion criteria listed and how best to ask sensitive questions to allay the fears and apprehension of participants*.* The research midwives will receive a monthly per diem.

## Results

Permission to conduct the study has been obtained from the University of Eastern Finland Committee on Research Ethics (Statement No. 13/2015) and the Ghana Health Service Ethics Review Committee (ID No: GHS-ERC:20/11/15), covering all the 10 proposed antenatal clinics. Application for funding to begin the trial is ongoing. Findings from the main trial are expected to be published by the end of 2019.

## Discussion

We propose an interventional study to fill the research gap on whether heavy lifting in pregnancy has negative effects on gestational age and birthweight, particularly among women in LMIC. There is no intervention study on the effects of lifting in pregnancy on birthweight and gestational age. Similar to many other health indicators, PTB and LBW prevalence rates underscore the overwhelming health disparities and inequalities between high-income and low-income countries. Strenuous physical activity, LBW, and PTB are common occurrences in low-income countries, which lack guidelines to protect against hazardous work conditions [11]. There are inconsistencies in the existing etiological studies on the subject, thereby creating an evidence gap [22]. As a result of the inconsistencies, recommendations and all other clinical guidelines lack the necessary evidential backing for practice. This clinic-level intervention using a stepped-wedge design will thus provide a vital contribution to the existing knowledge for the prevention of PTB and LBW, especially within LMIC.

Problems anticipated in the course of this intervention may arise from participant attrition, especially among controls, due to the longer duration of the trial. We intend to reduce attrition by explaining the potential benefits of the trial in detail and providing shopping vouchers, which is the third component of the intervention, to all control participants during postnatal periods. The strengths of the trial include the prospective data collection method, the study design, and the size of the study population. The results of this trial have the potential to justify the need for policy to modify the occupational and family environment of pregnant women within Ghana and other LMIC with similar socioeconomic conditions.

## Appendix

Multimedia Appendix 1 Proposed antenatal clinics and total number of antenatal attendance in 2017.

## Abbreviations

HIC: high income countries
LBW: low birthweight
LMIC: low- and middle-income countries
PTB: preterm birth
RCT: randomized controlled trial
