# Supplementary material for: A Liftless Intervention to Prevent Preterm Birth and Low Birthweight Among Pregnant Ghanaian Women: Protocol of a Stepped-Wedge Cluster Randomized Controlled Trial
Source: JMIR Res Protoc. 2018 Aug 23;7(8):e10095. doi: 10.2196/10095 (PMC6127499; doi:10.2196/10095)
Supplement: Multimedia Appendix 1 [file resprot_v7i8e10095_app1.pdf]

Multimedia Appendix 1. Proposed antenatal clinics and total number of antenatal attendance in 2017.

**No.**	**Name of Hospital/Clinic**	**Region (Location)**	**New ANC Registrants in 2017**	**Total ANC Attendance in 2017**
1	Achimota Hospital	Greater Accra	4448	19688
2	Atibie Government Hospital	Eastern	2227	6484
3	Cape Coast Teaching Hospital	Central	1009	7909
4	Dansoman Polyclinic	Greater Accra	974	3451
5	Kaneshie Polyclinic	Greater Accra	5366	21898
6	Komfo Anokye Teaching Hospital	Ashanti	10882	9439
7	Korle-Bu Teaching Hospital	Greater Accra	11854	18884
8	Legon Hospital	Greater Accra	605	2876
9	Mamprobi Polyclinic	Greater Accra	5149	17121
10	Ridge Hospital	Greater Accra	1423	13517
